# Complete mitochondrial DNA sequence of the East Asian minnow, *Pungtungia herzi* (Actinopterygii: Cypriniformes)

**DOI:** 10.1080/23802359.2020.1800423

**Published:** 2020-08-03

**Authors:** Teppei Taniwaki, Misaki Hiroshima, Mihiro Imakita, Yuu Kishimoto, Hisashi Okuyama, Jun-ichi Takahashi

**Affiliations:** a Osaka High School, Osaka, Japan; b Osaka High School Science Research Club, Osaka, Japan; c Faculty of Life Sciences, Kyoto Sangyo University, Kamigamo, Motoyama, Kita-ku, Kyoto, Japan

**Keywords:** Minnow, next-generation sequence, *Pungtungia herzi*, Yodo River

## Abstract

Some populations of the East Asian minnow *Pungtungia herzi* Herzenstein, 1892, which are naturally distributed in western Japan, have declined and are considered endangered. However, *P. herzi* has become a domestic invasive species in eastern Japan. Thus, knowledge of genetic features and phylogenetic relationships of *P. herzi* is important for conservation of this species and understanding its impact on ecosystems. We analyzed the complete mitochondrial genome using next generation sequencing of the East Asian minnow *P. herzi* from Yodo River, Osaka Prefecture, Japan. The mitochondrial genome of *P. herzi* consists of a circular molecule of 16,599 bp that includes 13 protein-coding genes (PCGs), 22 tRNA genes, two rRNA genes, and one control region. The heavy (H)-strand was predicted to have 12 PCGs, 14 tRNA, and two rRNA genes, while the light (L)-strand was predicted to contain one PCGs and eight tRNA genes. The average AT content was 57.68%. The genes ATP8 and ATP6, ATP6 and COIII, ND4L and ND4, and ND5 and ND6 shared seven, one, seven, and four nucleotides, respectively. The initiation codons ATG and GTG were found in 12 and one genes, respectively. The termination codons TAA, TAG, incomplete TA–, and single T–– were observed in nine, one, one, and two genes, respectively. All the tRNA genes possessed a cloverleaf secondary structure. The phylogenetic relationships inferred using 13 PCGs (based on the maximum likelihood) were consistent with previous studies that predicted interrelationships of Cypriniformes.

*Pungtungia herzi* Herzenstein, 1892 is known for parasitizing other fish species (e.g. Baba and Karino 1998; Baba 2010) and is distributed across the Korean Peninsula and Japan. In Japan, *P. herzi* is naturally distributed towards west from Fukui, Gifu, and Mie Prefectures (Yashima et al. 2011; Ohnaka and Mukai 2019). Populations of *P. herzi* have declined in some of these areas (e.g. Ito and Morimoto 2003; Osaka Prefecture 2014). However, *P. herzi* is a domestic invasive species, mainly in the Kanto region, and is a concern because of its influence on ecosystems (e.g. Yashima et al. 2011). Hence, understanding the genetic features and phylogenetic relationships between *P. herzi* populations and closely related species is important. Here, we present the complete mitochondrial genome of *P. herzi* from Osaka Prefecture, Japan, where it is being designated as an endangered species (Osaka Prefecture 2014).

*Pungtungia herzi* were captured alive from Yodo River, Osaka Prefecture, Japan, and stored in the Lake Biwa Museum (accession number: LBM-1210058083). DNA samples were immediately extracted using NucleoSpin Tissue (MACHEREY-NAGEL) and stored in a freezer at −20 °C for mitochondrial DNA analysis. Genomic DNA isolated from one fish was sequenced using the Illumina MiSeq platform (Illumina, San Diego, CA, USA). The complete mitochondrial genome of *P. herzi* (AB239598; Saitoh et al. 2006) was used as a reference sequence. The resultant reads were assembled and annotated using the MITOS web server (Bernt et al. 2013) and Geneious R9 (Biomatters) software. Thirteen protein-coding genes (PCGs) and two rRNA genes sequences were aligned using MEGAX (Kumar et al. 2018). The phylogenetic analysis was performed with the maximum likelihood (ML) criterion using TREEFINDER version of March 2011 (Jobb 2011).

We succeeded in sequencing the entire mitochondrial genome of *P. herzi* from Osaka Prefecture (DDBJ accession number LC519883). The genome resembles the genomic organization common in *P. herzi* and consisted of a closed loop that was 16,599 bp-long, which included 13 PCGs, 22 tRNA genes, two rRNA genes, and one control region. The average AT content was 57.68%. The heavy strand was predicted to have 12 PCGs, 14 tRNA, and two rRNA genes, while the light strand was predicted to contain one PCG and eight tRNA genes. Among the PCGs, *ATP8* and *ATP6*, *ATP6* and *COIII*, *ND4L* and *ND4*, and *ND5* and *ND6* shared seven, one, seven, and four nucleotides, respectively. Twelve PCGs of the *P. herzi* mitochondrial genome started with ATG, the *COI* gene started with GTG, and the termination codons TAA and TAG were observed in nine PCGs and the *ND4* gene, respectively. The incomplete stop codons TA (*COIII*) and T (*COII* and *Cytb*) were identified. All tRNA genes possessed a cloverleaf secondary structure. A phylogenetic analysis was conducted using the sequence information of 13 mitochondrial PCGs from 15 Japanese Gobioninae taxa (Figure 1). Two Japanese *P. herzi* formed a sister clade. The phylogenetic relationships of Japanese Gobioninae taxa were consistent with a previous study, which predicted interrelationships of the Cypriniformes (Saitoh et al. 2006; Tang et al. 2011).

**Figure 1.**
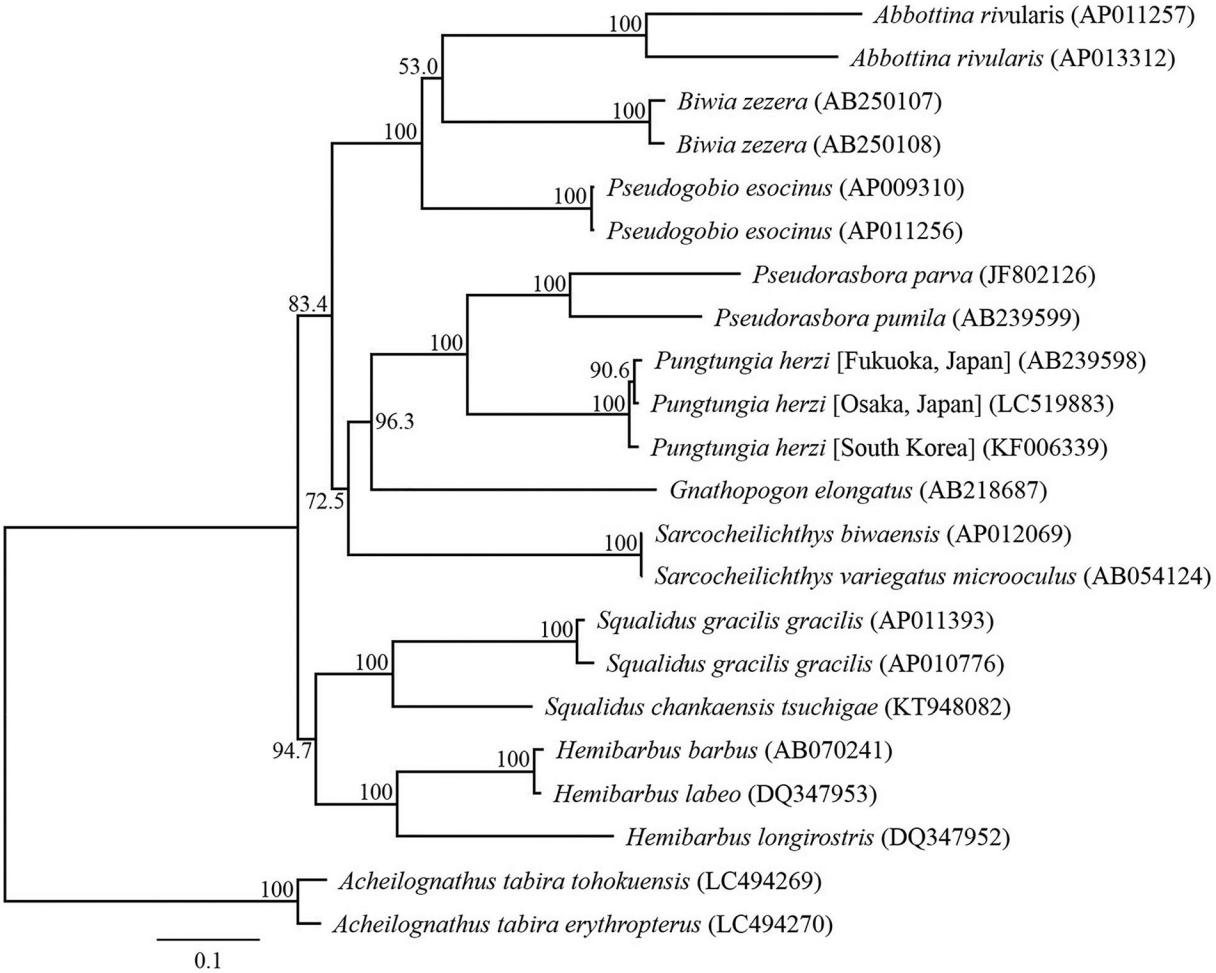
Phylogenetic relationships (maximum likelihood) of the Japanese Gobioninae taxa based on the nucleotide sequences of the 13 protein-coding genes of the mitochondrial genome. Sequences from *Acheilognathus tabira tohokuensis* (LC494269) and *A. tabira erythropterus* (LC494270) were used as an outgroup (Nagata and Kitamura 2019). These sequences were separated by codon positions, and for each partition, the optimal models of sequence evolution were used in the maximum likelihood method using TREEFINDER, based on the corrected Akaike information criterion. The numbers at the nodes indicate the bootstrap support inferred from 1000 bootstrap replicates. Alphanumeric terms indicate the DNA Database of Japan accession numbers.

## Data Availability

The data that support the findings of this study are openly available on GenBank using the accession numbers LC519883 (https://www.ncbi.nlm.nih.gov/nuccore/LC519883).
